# Comparison of Fracture Resistance in Thermal and Self-Curing Acrylic Resins—An In Vitro Study

**DOI:** 10.3390/polym13081234

**Published:** 2021-04-11

**Authors:** António Sérgio Silva, Aurora Carvalho, Pedro Barreiros, Juliana de Sá, Carlos Aroso, José Manuel Mendes

**Affiliations:** 1 Dental Science Department, Instituto de Investigação e Formação Avançada em Ciências e Tecnologias da Saúde (IINFACTS), Rua Central da Gandra 1317, 4585-116 Gandra, Portugal; carlos.ribeiro@iucs.cespu.pt (C.A.); jose.mendes@iucs.cespu.pt (J.M.M.); 2 Department of Oral Rehabilitation, Instituto Universitário de Ciências da Saúde Rua Central da Gandra 1317, 4585-116 Gandra, Portugal; a8690@alunos.cespu.pt (A.C.); jose.fonseca@iucs.cespu.pt (P.B.); juliana.sa@iucs.cespu.pt (J.d.S.)

**Keywords:** acrylic resins, fracture resistance, self-curing, thermosetting

## Abstract

Thermal and self-curing acrylic resins are frequently and versatilely used in dental medicine since they are biocompatible, have no flavor or odor, have satisfactory thermal qualities and polishing capacity, and are easy and fast. Thus, given their widespread use, their fracture resistance behavior is especially important. In this research work, we comparatively analyzed the fracture resistance capacity of thermo and self-curing acrylic resins in vitro. Materials and Methods: Five prosthesis bases were created for each of the following acrylic resins: Lucitone®, ProBase®, and Megacryl®, which were submitted to different forces through the use of the CS^®^ Dental Testing Machine, usually mobilized in the context of fatigue tests. To this end, a point was defined in the center of the anterior edge of the aforementioned acrylic resin bases, for which the peak tended until a fracture occurred. Thermosetting resins were, on average, more resistant to fracture than self-curable resins, although the difference was not statistically significant. The thermosetting resins of the Lucitone® and Probase® brands demonstrated behavior that was more resistant to fracture than the self-curing homologues, although the difference was not statistically significant. Thermosetting resins tended to be, on average, more resistant to fracture and exhibited the maximum values for impact strength, compressive strength, tensile strength, hardness, and dimensional accuracy than self-curing resins, regardless of brand.

## 1. Introduction

Acrylic resins are organic compounds derived from ethylene (polymers) and result from the reaction between methylpolymethacrylate and methylmethacrylate, normally supplied in powder form and in liquid form, respectively. A polymerization reaction (combination of both compounds) is a union of monomers in a macromolecule, which is a malleable and moldable mass [1,2]. The polymerization reaction can result from thermal or chemical activation. Thus, thermosetting acrylic resins are thermally activated, hardening permanently when exposed to certain temperatures, becoming more resistant and dimensionally stable, and not reacting to subsequent re-heating. In turn, self-curing acrylic resins are chemically activated: they can be polymerized at room temperature and require a chemical activator (a tertiary amine: di-methyl-para-toluidine) [2].

Acrylic resins are versatile and frequently used in dentistry, for example, in complete dentures, temporary restorations, and even prosthodontic implant rehabilitations [3]. This is possible because they tend to be biocompatible, have no taste or odor, have satisfactory thermal qualities, can be polished, and are easy and quick to repair. However, the lack of marginal adaptation or roughness of this type of polymer can cause irritation and inflammation, contributing to the accumulation of biofilm [4,5]. The toxicity of acrylic resins is fundamentally due to the release of methacrylate monomers and is different according to their structure. When the polymerization time is increased, the amount of residual monomers is reduced, also decreasing secondarily the probability of occurrence of cytotoxic effects. An incubation of 7 h in water at 70 °C has been recommended, followed by an incubation of 1 h at 100 °C, causing the conversion of the monomer into polymer. Boiling acrylic resins in the polymerization phase for at least 30 min at maximum temperatures has also been suggested, as well as immersing the thermopolymerizable prosthetic bases in water for 1 to 2 days prior to delivery [4,5]. In addition, fundamental questions have been raised about their properties regarding chemical and dimensional stability, resistance, and longevity [1,4], which must be explored. These are characterized by low tensile strength (from 27.5 to 82.7 MPa), low flexion (from 62.1 to 103.5 MPa), and low impact resistance (Charpy test results between 0.098 to 1.27 J were obtained) [1]. Clinically, this is reflected in 10% of acrylic prostheses fracturing in the first three years of use [6]. In addition, factors such as porosity, presence of residual monomer, presence of microfractures or cracks, or poor muco-support adaptation of the removable prosthesis to the residual crest make them particularly susceptible to fracture [7]. Thus, since a larger amount of residual monomer is identified in chemically activated acrylic resins compared with thermally activated ones, it is possible to suppose a greater propensity for fracture for the former due the continuous polymerization and contraction of the acrylic [8]. Previous studies already identified the greatest resistance to deformation and fracture by thermally activated acrylic resins [9].

To avoid fractures, acrylic resins’ structures have been modified through the use of, for example, co-polymers and binding agents [3,10]. Fiber glass, aramid, or nylon have been introduced to increase the fracture resistance or the elasticity module of polymeric materials [11], and new processing and activation techniques have been developed toward the same goal [7]. These investments, both in research and in practice, emphasize the relevance of the topic.

The fatigue of the acrylic resin results from a continuous process of forces application that causes permanent deformation. Thus, the material ability to withstand masticatory tensions and absorb energy [1] is the main resistance factor to fracture. Microstructural behavior, surface defects, and fracture onset sites can provide more information about the fracture resistance of thermo and self-curing acrylic resins [6,7]. As such, the purpose of this study was to evaluate fracture resistance between thermo and self-curing acrylic resins; this is crucial because wear resistance of denture teeth has been considered one of the most important requirements for oral rehabilitation of edentulous patients with removable dentures, in order to maintain a stable occlusal support over time. Wear of the occlusal surfaces may result in insufficient posterior tooth support, loss of chewing efficiency, and nonfunctional activities. Although wear of acrylic resin teeth has also been related to the loss of vertical dimension of occlusion with complete dentures, the major factor affecting it is the reduction of residual ridges by resorption [1,7].

## 2. Materials and Methods

### 2.1. Sample Characteristics

The acrylic resins used were selected for their relevance and usefulness in dentistry and for their stability under normal conditions of use and storage.

The sample of self-curing acrylic resins was composed of:

- Megacryl S + N^®^ (Megadental, Büdingen, Germany): Composed of acrylic based on methyl-methacrylate, without tertiary amine or cadmium. Excellent fluidity, mechanical properties, natural coloring, and safe behavior, so it is stable and modellable;
- ProBase Cold^®^ (Ivoclar Vivadent, Zurich, Switzerland): Composed of acrylic based on methyl-methacrylate. Excellent fluidity, mechanical properties, and safe behavior, so it is stable and modellable; the polymer composition is >95% polymethylmethacrylate; and
- Lucitone HIPA^®^ (Dentsply Sirona, Ballantyne Corporate Pl, Charlotte, NC, USA): Composition based on methyl-methacrylate (70–90%).

The sample of thermopolymerizable acrylic resins was composed of:

- Megacryl Hot + Don^®^ (Megadental, Büdingen, Germany): Composed of acrylic based on methyl-methacrylate, suitable for the compression and injection process. Excellent fluidity, mechanical properties, natural coloring, and safe behavior, so it is stable and modellable;
- ProBase Hot^®^ (Ivoclar Vivadent, Zurich, Switzerland): Composed of acrylic based on methyl methacrylate (50–100%); excellent fluidity, mechanical properties, and safe behavior, so it is stable and modellable. The polymer composition is >95% polymethylmethacrylate;
- Lucitone 199^®^ (Dentsply Sirona, Ballantyne Corporate Pl, Charlotte, NC, USA): Composition based on methyl-methacrylate (80–100%).

### 2.2. Data Collection

A standard laboratory protocol was established and applied at the Institute for Research and Advanced Training in Health Sciences and Technologies (IINFACTS-CESPU, Gandra, Paredes, Portugal) to test all selected samples.

**Figure 1:**
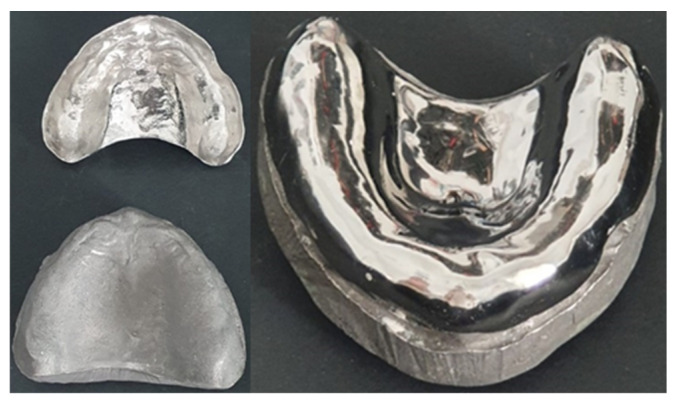
Prefabricated model.

**Figure 2:**
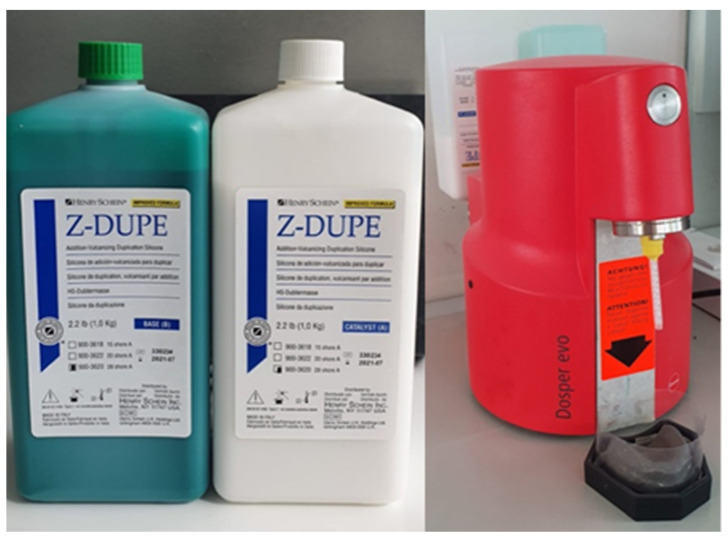
Z-Dupe 28 shore A silicone (base + catalyst) and Dosper Evo duplication machine.

**Figure 3:**
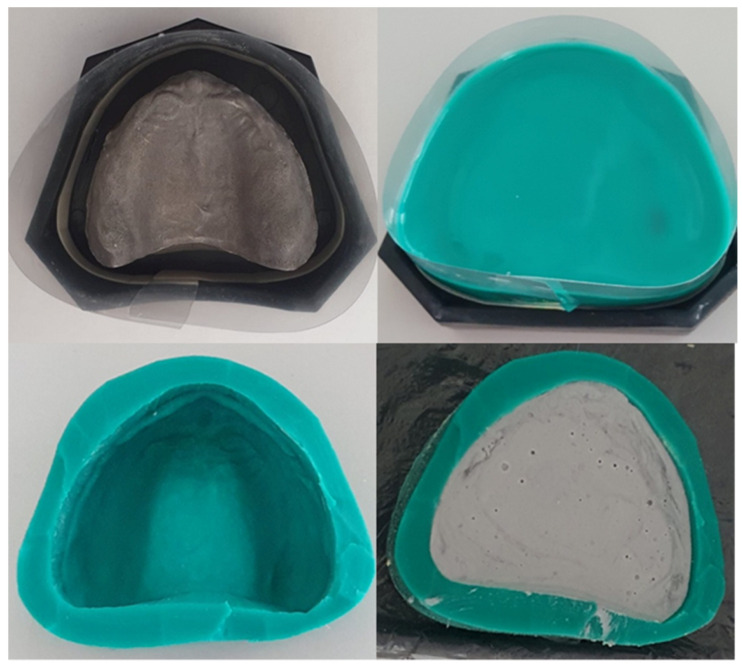
Casting process of the replicated model.

**Figure 4:**
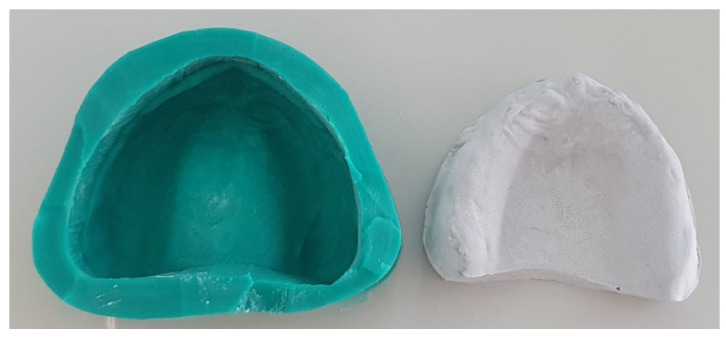
Replicated model.

For models fabrication:

1. The preparation for the experimental phase started with the multiplication of 30 units of the prefabricated model (Figure 1), in type IV plaster.

2. The multiplication of the prefabricated mold was carried out with a Dosper Evo (Dreve Dentamid GmbH, Unna, Germany) duplicating machine using the Z-Dupe 28 shore A silicone (base + catalyst) (Henry Schein, Melville, NY, USA) (Figure 2).

3. The prefabricated mold was removed and the cooping was poured to type IV plaster (Figure 3 and Figure 4).

4. We created 5 bases of each acrylic resin described above, with dimensions of 60 × 45 mm^2^ and height of 2 mm using the prefabricated models.

**Figure 5:**
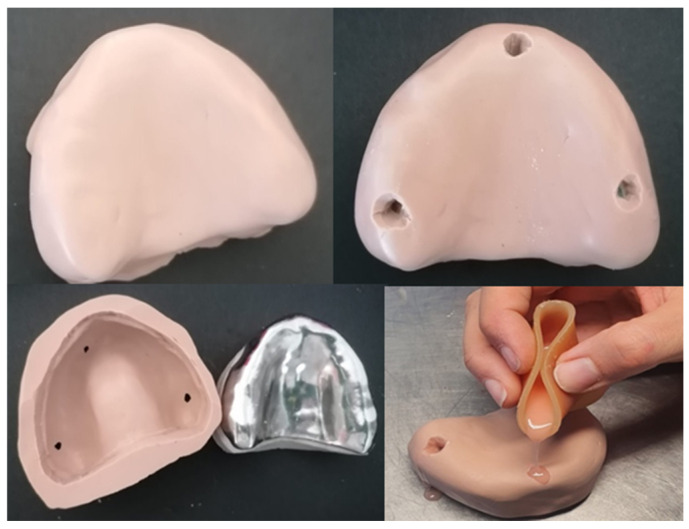
Self-curing acrylic bases fabrication.

For fabrication of self-curing acrylic bases:

1. We developed a silicone wall covering a mold with the metallic base. Once it hardened, it was separated, and a hole was made in the silicone.
2. To pour the acrylic, the metal base was removed.
3. The model was isolated with plaster insulator, and the silicone wall was placed on top of the same model (Figure 5).

4. We created the base using acrylic resin according to the manufacturer’s recommendations, pouring it between the wall and the mold and placing them in a pressure-cooker at 2 bars for the recommended time and temperature.

**Figure 6:**
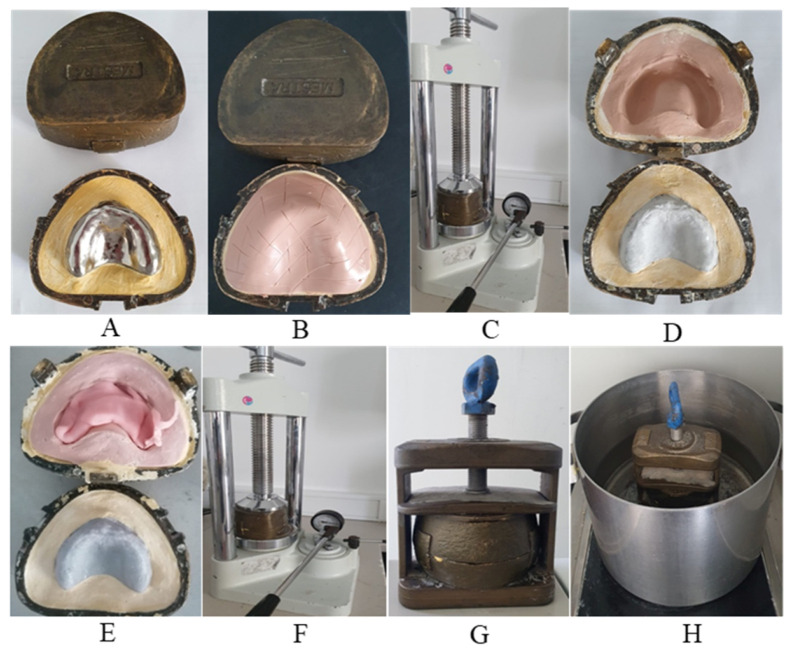
Thermopolymerizable acrylic bases fabrication. (**A**) Metal base in muffle; (**B**) silicone wall; (**C**) counter muffle closed with type III plaster; (**D**) mold of metal base; (**E**) placing the acrylic between the mold and the wall; (**F**) closing the muffle with the help of the press; (**G**) press used; (**H**) pan with hot water following the manufacturer’s time and temperature recommendations.

For fabrication of thermopolymerizable acrylic bases:

1. We placed the model with the metal base in muffle and the formation of a silicone wall on top of that same base.
2. The counter muffle was closed with type III plaster and placed in the press until the plaster hardened. Subsequently, the muffle was opened, removing the metal base (Figure 6), and the acrylic was produced based on the supplier’s instructions.

3. We placed the acrylic between the mold and the wall, closing the muffle again with the help of the press (Figure 6), placing them in a pan with hot water following the manufacturer’s time and temperature recommendations.

All bases were numbered (I, II, III, IV, V, and VI) on the posterior edge of the first quadrant. All these bases were subsequently subjected to the same vertical force through the use of the CS^®^ Dental Testing Machine (Table 1) (Idearum, Igualada, Barcelona, Spain), normally used in the context of fatigue tests; all bases were numbered (I, II, III, IV, V, and VI) on the posterior edge of the first quadrant. All these bases were subsequently subjected to the same vertical force through the use of the CS^®^ Dental Testing Machine (Idearum, Igualada, Barcelona, Spain), normally used in the context of fatigue tests, built in agreement with 2006/42/CE safety of machines and the norms EN 12100-1/2, EN 954-1, EN 1037, EN 61310-1/2, EN 60204-1, EN ISSO 14121-1, and EN ISSO 13850. 

**Figure 7:**
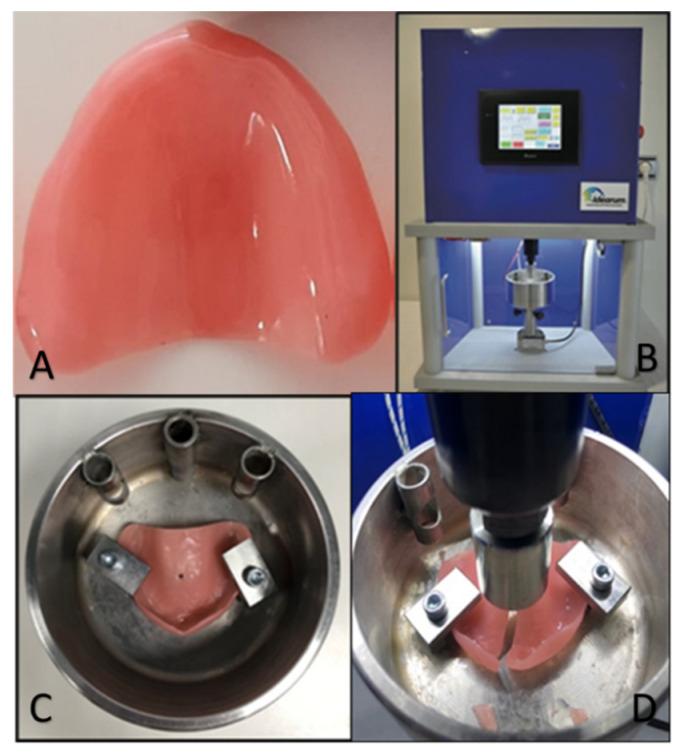
Examples of (**A**) acrylic base (control); (**B**) CS^®^ Dental Testing Machine; (**C**) support of the acrylic base; (**D**) simulated movement of dynamic mastication forces.

Subsequently, a point was defined in the center of the anterior edge of the base of the referred resins toward which the peak tended until a fracture occurred (Figure 7). The fracture force was measured and systematized by the load cell, and this information was directly transferred to Microsoft Office Excel (Microsoft, Redmond, Washington, DC, USA) software.

### 2.3. Statistical Analysis

Data were analyzed in SPSS^®^ (IBM, Armonk, NY, USA), version 24. First, exploratory data analysis was performed, which detected one outlier, Probase^®^. However, as this value was within the limits of the observations of the other brands, it was included in the analysis.

The variable (force) was then described using the mean (M) and standard deviation (SD) for the fracture torques, expressed in Kgf, after evaluating the asymmetry coefficient (<|1|) and the histograms.

The normality of the distributions was assessed using the Shapiro–Wilk test, used for *n* < 50, confirming the necessary assumption for the use of parametric tests for the distributions of the three brands under study. The homogeneity of variances was evaluated and confirmed with the Levene test.

The comparison of the average fracture torque by brand and type (auto and thermopolymerizable) was performed with two-way ANOVA, with calculation of the *F* test for brand, type, and brand × type interaction. The effect size was also calculated using eta2 (η^2^), considering as cutoff points slight (0.01), moderate (0.06), and high (0.14) effects.

The *t*-test for one sample was used to compare the six groups of brand × type samples by the reference limits of the human bite of 6 and 8 Kgf.

The maximum level of significance considered was 5%.

**Figure 8:**
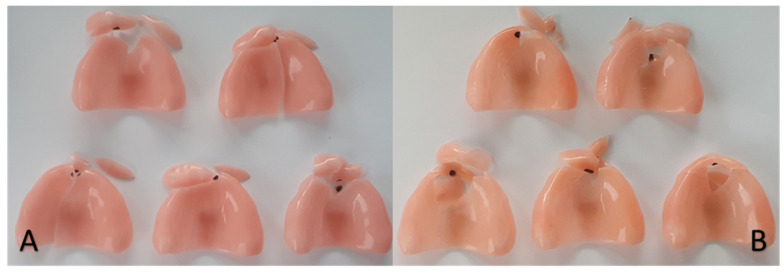
ProBase Cold^®^ (**A**) and ProBase Hot^®^ (**B**).

**Figure 9:**
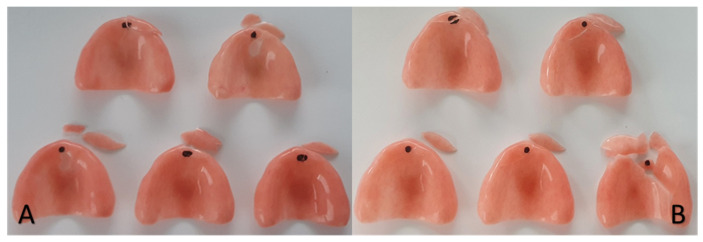
Lucitone HIPA^®^ (**A**) and Lucitone 199^®^(**B**).

The ProBase Cold^®^ and ProBase Hot^®^ brands consecutively presented the greatest fragment loss during the fracture (Figure 8). The Lucitone HIPA^®^ and Lucitone 199^®^ brands were the ones with the lowest number of fragment losses (Figure 9).

The peak of the fracture zone was the place where all the bases shattered the most, with only 5 of the 30 bases fracturing in the middle.

Notably, none of the plates tested had porosities.

## 3. Results

We evaluated 30 samples, 10 from each brand—Lucitone^®^, Megacryl^®^, and Probase^®^—with the objective of testing the resistance of self- and thermopolymerizable resins by evaluating the maximum fracture torque, expressed in Kgf. All measurements were greater than 15 Kgf and less than 30 Kgf. The global mean of the fracture torque was 21.89 (SD = 3.46), with a minimum of 15.72 and a maximum of 29.48.

Table 2 shows the results for the two-way ANOVA in the comparison of the average fracture torque values by brand and type of resin. No statistically significant results were found in the comparison by brand (*F* (2.24) = 1.92; *p* = 0.169), type (*F* (1.24) = 2.04; *p* = 1.66), and brand interaction × type (*F* (2.24) = 2.28; *p* = 0.124). The effect sizes found were high, mainly in the comparison between brands (η^2^ = 0.14), where the Megacryl^®^ and Lucitone^®^ brands stood out, with an average fracture resistance of 22.78 (SD = 2.84) and 22.96 (SD = 3.48), respectively, both of which were higher than Probase^®^. The effect size was also found high in the interaction of the brand with the fracture (η^2^ = 0.16), where we observed that among the three brands, Lucitone^®^ was the only one that obtained a relevant gain in terms of fracture torque with the type of thermosetting resin (M = 25.49; SD = 2.68) compared with the self-curing type (M = 20.42; DP = 2.00). At this level, the average global result of the Lucitone^®^ brand was obtained at the cost of a value very close to the minimum in the self-curing type and the highest value of the entire study in the thermal-curing type, whereas Megacryl^®^ and Probase^®^ did not undergo substantial changes between the two types.

**Figure 10:**
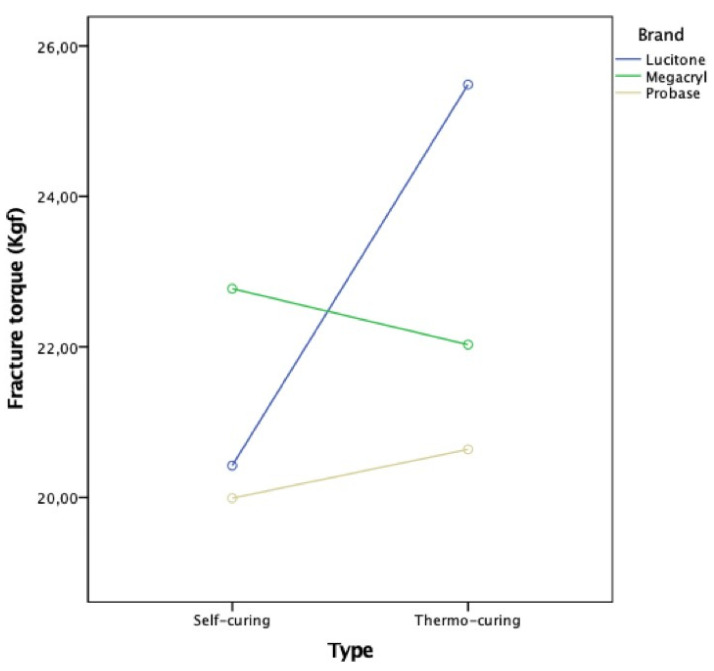
Fracture torque evaluation: brand/resin type

Figure 10 corroborates the results in Table 2. The Megacryl^®^ brand stood out in the self-curing type, with a small reduction in the average fracture torque in the thermo-curing type. The Lucitone^®^ brand stood out in the evolution between the auto and thermopolymerizable types. Probase^®^ achieved the lowest results of all brands.

**Figure 11:**
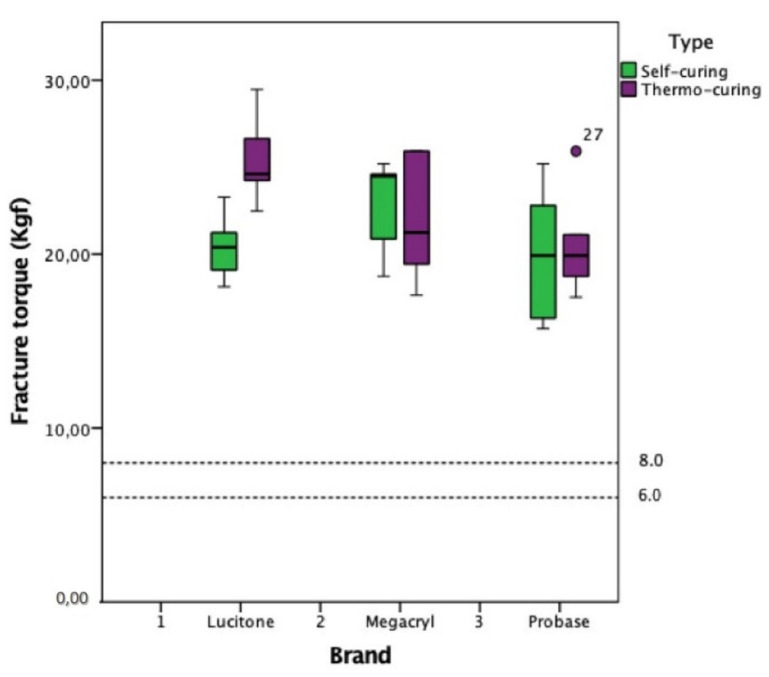
Box and line diagrams for the distribution of fracture torque by brand and type of resin (6 and 8 Kgf comparison).

All observations were above the limits of 6 Kgf and 8 Kgf (Figure 11). Statistically significant results were observed, with a maximum error of 0.3% in comparison with the reference limits of 6 and 8 Kgf (Table 3), suggesting that the evaluated types of resin are well above the limit for human dynamic masticatory forces.

## 4. Discussion

In dentistry, the quality of products provided to patients has an important impact on patient quality of life. For acrylic resins, fracture resistance is a main theme because of the increased costs involved in the repair of acrylic prostheses. The prostheses may fracture due to fatigue caused by prolonged wear and degradation of the material or by the excessive masticatory load, passing the plastic phase of the material. Therefore, knowing the clinical performance of different acrylic resins with regard to fracture behavior can indicate how to avoid fractures, avoiding potentially unnecessary costs and improving the quality of life of patients [9,10].

In this study, we aimed to comparatively analyze, in vitro, the fracture resistance capacity of thermal and self-curing acrylic resins. From the main results of the study, we did not find statistically significant differences between the brands tested or in the brand × type interaction. The results refute our hypothesis that there are differences in fracture resistance between the thermo and self-curing acrylic resins. We found that the most resistant brands were Megacryl® and Lucitone®, the former being more stable to fracture resistance regardless of the type of resin. The Probase® brand was the least resistant to fracture, both for self- and thermal-curing resins [11,12].

The literature indicates that self-curing resins tend to be less resistant to fracture because of the larger amount of residual monomer they tend to generate [13,14]. Some studies proved this phenomenon [10]. Even though our results did not allow us to identify significant differences in the fracture resistance values of self- and thermosetting acrylic resins, some tendency toward a lower propensity for fracture in thermosetting acrylic was found, which agrees with the literature [11,12,15].

Regarding the resistance of the different acrylic resins, different studies report that there should be no bubbles or voids in the material for the prosthesis base when seen without application. In addition, porosity values greater than 11% have been associated with reduced mechanical properties, impaired appearance, and retention of liquids and microorganisms [16,17].

The fracture resistance of acrylic resins is also related to mechanical properties such polymerization efficiency and the consequent creation of short polymer chains with low molecular weight [18,19]. Thus, the polymerization efficiency of the thermosetting resins used in the study may have been higher and thereby generated less residual monomer compared with normal. In addition, the elution of components and degradation products must be considered when assessing acrylic resin fracture resistance behavior in the mouth [20,21], noting that certain factors are not replicated in vitro, such as the effect of saliva pH (lower pH decreases fracture resistance) and different masticatory forces and directions. It is also important to consider that the brands tend to improve their products, which may affect the results obtained [22]. Sá J. et al. (2020) found that a low pH reduces the strength of acrylic resin, regardless of the processing technique. They concluded that after exposure to an environment of pH = 7, a higher average force to fracture could be sustained. [22]. Other authors also found a significant interaction between the brand and the pH: Low module of elasticity produces a larger resilience module; consequently, the energy-absorbing capacity is higher and the deflection force released on the material will be higher [18,19]. This result showed that the concentration of added elastomer was directly proportional to the resistance to impact. The addition of elastomer to the material increases its ability to absorb energy and can overcome the possibility of resin fracture, which can result in a prosthetic device being less susceptible to mechanical failure.

The occlusion strength in the centric position of patients with complete dentures is variable—according to a recent study, between 6 kgf and 8 kgf on average [23,24]. The types of acrylic resin evaluated are well above the limit for human occlusion strength. This is a finding to consider when choosing an acrylic resin for the creation of prostheses, a consideration which may be further developed in future in investigations in a real-world context.

The results are in line with the literature on the subject, but with a few significant differences to report. The fracture resistance behaviors of the acrylic resins discussed here and comparisons with other materials, such as thermoplastics, are important to investigate more deeply in the future. Furthermore, it is important to consider the introduction of strengthening modifications in the structure of acrylic resins through the use of, for example, co-polymers and binding agents [25,26,27], as well as to consider the development of new processing and activation techniques for the same purpose [28,29,30]. 

As described by Zafar MS (2020), many variety of fibers have been added and extensively characterized to improve acrylic properties such as carbon, which provides enhancement of the mechanical properties, including tensile strength, flexibility, fracture resistance; elastic modulus, aramid, which has good wettability and improved mechanical properties, such as fracture resistance; nylon, which improves flexural strength and structural elasticity and fracture resistance; or even glass, which provides excellent reinforcement and aesthetics compared with other fibers—a remarkable increase in the denture base toughness, Vickers hardness, impact strength, and flexural strength is observed, with a remarkable reduction in the deformation [31]. Another introduced option using computer-aided design and computer-aided manufacture (CAD/CAM) for producing removable dentures definitely addresses this demand, as both have fundamentally changed the manufacturing process. Instead of manually mixing the resin powder and liquid and then submitting the immersion to an arbitrarily chosen curing protocol, the poly(methyl methacrylate) (PMMA) resin blocks for CAD/CAM denture bases are industrially fabricated [32] and cured under “great heat and pressure” [32]. Therefore, it has been assumed and reported that the CAD/CAM denture base resins are highly condensed and have fewer micro-porosities [32]. This, in consequence, would mean that CAD/CAM denture base resins could have superior mechanical properties, which is probably why some of the CAD/CAM denture manufacturers advertise that their products have a very low minimum material thickness and high fracture resistance. Steinmassl et al. (2018) performed fatigue tests on a total of 80 standardized, rectangular CAD/CAM denture base resin specimens from five different manufacturers (AvaDent, Baltic Denture System, Vita VIONIC, Whole You Nexteeth, and Wieland Digital Dentures) and compared them with heat-polymerising resin and an autopolymerising resin. They concluded that base resins for CAD/CAM dental prostheses did not clearly demonstrate better mechanical properties than manually processed resins [32]. Thus, it is important that future investigations examine the topic in a more expanded way, seeking to generate complex and multifactorial comparative analyses of the various options, promoting robust conclusions about the potentialities and limitations of each option, and therefore supporting evidence-based practice.

## 5. Conclusions

The following conclusions were achieved:

(1) Thermopolymerizable resins were, on average, more resistant to fracture than self-curable resins, although the difference was not statistically significant. The thermopolymerizable resins of the Lucitone® and Probase® brands demonstrated behavior that was more resistant to fracture than the self-curing homologues, although the difference was not statistically significant.
(2) Thermopolymerizable resins more resistant to fracture were the Lucitone^®^ brand, followed by Megacryl^®^ and Probase^®^.
(3) Self-curing resins more resistant to fracture were those of the Megacryl^®^ brand, followed by Lucitone^®^ and Probase^®^.
(4) Megacryl^®^ had the most stable fracture resistance behavior regardless of the type of resin.
(5) The behavior and resistance of the resins evidenced in the study were well above the reference limit for the average human mastication force.

## Figures and Tables

**Table 1:** Technical Features—CS® Dental Testing Machine.

Technical Features—CS^®^ Dental Testing Machine		
Weight		49 kg
Maximum actuator power		0.2 kW.
Maximum actuator pair		0.64 N·m
Maximum advance speed of the actuator		3000 rpm
Actuator operating speed		1.06 mm/s
Power circuit		400 V AC
Maneuvering Circuit		230 V AC
Sound pressure level		L_eqA_ < 70 dB
Engine	Load cell	General
Course: 150 mm	Precision: 0.01 N	Touch screen
Maximal strength: 1600 N	Maximal strength: 300 N	USB Input
Speed: 210 mm/s		Electric power

**Table 2:** Bifactorial ANOVA for the comparison of the average values of fracture torque by brand and type of resin.

	Type			Statistical Tests		
Brand	Self-Curing (*n* = 5)	Thermo-Curing (*n* = 5)	Total (*n* = 10)	Brand	Type	Brand × Type
Lucitone^®^	20.42 (2.00)	25.49 (2.68)	22.96 (3.48)	*F*_(2.24)_ = 1.92 *p* = 0.169 η^2^ = 0.14	*F*_(1.24)_ = 2.04 *p* = 0.166 η^2^ = 0.08	*F*_(2.24)_ = 2.28 *p* = 0.124 η^2^ = 0.16
Megacryl^®^	22.78 (2.84)	22.03 (3.77)	22.40 (3.17)			
Probase^®^	19.99 (4.08)	20.64 (3.24)	20.32 (3.49)			
Total (*n* = 15)	21.06 (3.13)	22.72 (3.68)				

**Table 3:** Results of *t*-tests for the comparison of fracture torque with the reference limits of 6 and 8 Kgf.

	Self-Curing		Thermo-Curing	
Brand	Reference = 6 Kgf	Reference = 8 Kgf	Reference = 6 Kgf	Reference = 8 Kgf
Lucitone^®^	*t*(4) = 16.16 (*p* < 0.001)	*t*(4) = 13.92 (*p* < 0.001)	*t*(4) = 16.28 (*p* < 0.001)	*t*(4) = 14.61 (*p* < 0.001)
Megacryl^®^	*t*(4) = 13.23 (*p* < 0.001)	*t*(4) = 11.65 (*p* < 0.001)	*t*(4) = 9.51 (*p* = 0.001)	*t*(4) = 8.32 (*p* = 0.001)
Probase^®^	*t*(4) = 7.66 (*p* = 0.002)	*t*(4) = 6.56 (*p* = 0.003)	*t*(4) = 10.09 (*p* = 0.001)	*t*(4) = 8.71 (*p* = 0.001)

## Data Availability

No data other than that shown in the manuscript has been reported.
